# UAV Visual and Laser Sensors Fusion for Detection and Positioning in Industrial Applications

**DOI:** 10.3390/s18072071

**Published:** 2018-06-28

**Authors:** Edmundo Guerra, Rodrigo Munguía, Antoni Grau

**Affiliations:** 1 Department of Automatic Control, Technical University of Catalonia UPC, 08034 Barcelona, Spain; edmundo.guerra@upc.edu; 2 Department of Computer Science, CUCEI, University of Guadalajara, Guadalajara 44430, Mexico; rodrigo.munguia@upc.edu

**Keywords:** Unmanned Autonomous Vehicle, pose determination, LiDAR registration, apparent contour

## Abstract

This work presents a solution to localize Unmanned Autonomous Vehicles with respect to pipes and other cylindrical elements found in inspection and maintenance tasks both in industrial and civilian infrastructures. The proposed system exploits the different features of vision and laser based sensors, combining them to obtain accurate positioning of the robot with respect to the cylindrical structures. A probabilistic (RANSAC-based) procedure is used to segment possible cylinders found in the laser scans, and this is used as a seed to accurately determine the robot position through a computer vision system. The priors obtained from the laser scan registration help to solve the problem of determining the apparent contour of the cylinders. In turn this apparent contour is used in a degenerate quadratic conic estimation, enabling to visually estimate the pose of the cylinder.

## 1. Introduction

For a long time now robots and automata can be found in industrial and civilian operations as part of complex systems presenting some degree of automation. The introduction of these technologies has been driven by several factors, mainly the increased efficiency derived from the automation, but also the better and safer conditions for human employees. In many areas, the introduction of these technologies has been delayed because there is still need of human hard to reproduce capabilities. One of these capabilities, characteristic to human beings, is the generality and adaptability of human response, which makes them especially suited for supervisory and monitoring tasks.

Monitoring and maintenance tasks rely heavily in availability of the information, which can be obtained from remote/installation sensors, but many times require actual physical inspection of some elements. This is especially true for industry, were it would be impossible to sensorize all the elements/points which must be inspected or monitored at some time as part of the maintenance operations. An example of such elements would be pipes and canalizations, especially in heavy industries, where kilometers of pipes have to be periodically inspected.

These pipes and tubes are common structures not only in industry but also in urban environments, and can be frequently found in hard to reach areas. This poses a problem for the mentioned monitoring and maintenance operations, as those operations are commonly performed by human personnel or ground-based unmanned vehicles (UGVs) with great efficiency, become expensive and exceptionally risky in inaccessible areas. In such scenarios, operations with humans in high and/or hard to reach areas generally imply shutting off ordinary operation, building temporary scaffolds, and following complex safety protocols and procedures to minimize risks. In these situations, any opportunity to reduce the participation or risks taken by human personnel can have a great impact, both economically and in safety terms.

In this context, Unmanned Aerial Vehicle (UAV)-based solutions have started to appear. While UAV drones have been present for a long time, developments in microelectromechanical systems (MEMS) and battery technologies have produced an explosive growth of the field. This has made them cheaper and easier to deploy, with a big research and development community supporting them, especially for the massively popular rotary-wing multicopters. This kind of UAV has already a strong presence in the audiovisual production and the surveying industry, and is gaining a foothold in other industries.

On the other side, the problem of locating pipes from robotic platforms is not new. There are many works that try to locate defects in pipes from the inside [1,2] which use robots that try to build a map of the pipe while they navigate inside the pipe. Those systems are based mainly in odometry and inertial measurement units to build the path and the map to locate themselves [3]. However, authors are not facing this kind of problem in this research. In [4], the authors present an on-board UAV visual system which tries to avoid collisions of such flying robots. The range visual detection varies depending on the detected elements but the accuracy in objects detection is not enough to locate the UAV with enough precision to obtain a good pose of the robot. In [5] another obstacle detection-equipped UAV is presented. The robot has visual systems, laser, barometer and ultra-sound on-board and, although they use a PTAM scheme [6] to locate it, the excess in sensors means that the accuracy in fusion gives large errors in location; at low heights the barometer is unreliable due to turbulences, and at heights above 5 m the ultrasonic distance sensor drops out.

The objective of this work is to develop a multimodal solution to detect and localize pipes from an UAV in order to enable inspection and monitoring operation in industrial environments inaccessible to humans. An initial attempt to produce independent solutions with monocular cameras and Light Detection and Ranging (LiDAR) sensors, and evaluate which provided a better solution led to the development of several of the methods described. Section 3 describes the methods developed for the LiDAR, detailing two different architecture optimized for accuracy and for performance respectively. Section 4 describes the methods developed for the monocular camera, including a brief discussion of the pose recovery method adopted. After the discussion in Section 3 and Section 4 about the weaknesses and strengths of each of the methods developed, the integrated multimodal solution proposed is detailed in Section 5. The experimental results obtained to evaluate the different methods are detailed in Section 6, after a brief description of the different experimental setups used.

## 2. UAV Architecture and Properties

Unlike ground robots, one of the most challenging aspects of robotics in the context of UAV is the limited payload. The equipment deployable on board is strictly limited by weight, its own and that of the batteries required to power the device. This fact is translated into very limited computational power deployable on board, even introducing additional single board computers (SBCs). The chances of delegating computational efforts to other systems are also constrained by the range, bandwidth and latency of wireless communications; so the general assumption is to deploy anything needed at real-time performance on-board.

This affects the architecture of the robotic UAV, not only in hardware terms, but also from a high level architecture point of view. Thus, the common approach of deploying a single computing unit in the form of the Flight Management Unit (FMU) is ignored in favour of deploying an additional SBC. This additional computing unit will be responsible of all the hardware and processes not needed in the low-level control loops to guarantee UAV stability and safety. The FMU will receive data from those sensors, which require low computational power to process it (GPS, inertial and height sensors, etc…) and control the low-level operation of the UAV. This way, the heaviest computational task, such as image processing, localization in maps, video streaming and communications, etc. are delegated to the SBC.

**Figure 1:**
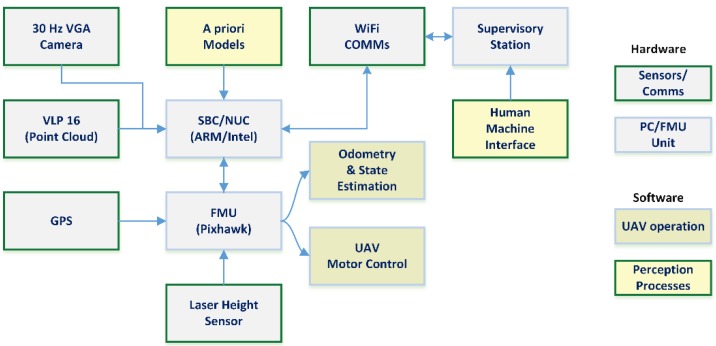
Architecture diagram with main sensors and processing hardware, noting some relevant processes for this work.

Figure 1 shows the architecture of the UAV drones considered in this work. Though the architecture was initially developed using Odroid SBCs (based on ARM processors, roughly equal to a high-end smartphone), the kind of computational power required by the proposed approaches required upgrading the hardware to an Intel Next Unit of Computing (NUC) device (offering the same performance as a mid-to-high end laptop).

Under this architecture, the FMU is still responsible of the odometry estimation, so the research and experiments have to account for the error characterization in these measurements.

## 3. LiDAR-Based Detection and Segmentation of Cylinders

Detection and positioning of pipes using LiDAR or similar range finder sensors is essentially a problem of shape detection in point clouds. There are many approaches to this problem, but they are generally based on five wide categories: edge based, region based, attribute based, graph based or model based methods. Each category shares a wide set of features, according to its procedures and strategies.

Edge based methods try to find the edges of a region of similar points, generally through identification of those points presenting a rapid divergence of the metric with respect to the neighbors. Some methods are based in gradient techniques [7], while other detect different edges and group them, producing scan lines representing surfaces [8]. Approaches like the latter one are suitable for only-range sensors, but produce weak results when the point-cloud density is uneven. On the other hand, region-based methods use local neighborhood information to build regions of points with similar features, and isolate regions according to the dissimilarity, thus growing regions instead of delimiting them as the edge-based methods. Though they have been reported to provide better results than edge-based methods, they have low accuracy determining the limits of the regions, and can require accurate seeds to start growing regions [9].

Methods based on attributes, like [10], work as a two-step process: in the first step an attribute or set of attributes is computed; and in the second step the data points are classified (commonly through clustering) according to the attribute. Though they are resilient and the clustering can be used to introduce clues, they are largely dependent on the attribute that was chosen and its selection is not a trivial problem.

Graph-based methods read the whole point-cloud as a graph, with the simplest case matching each point to a node. They can produce very good results, as they can benefit from many techniques commonly applied to graph-based problems, like Markov Random Fields [11], k-nearest neighbor (kNN) [12], or conditional random fields (CRF) [13], to cite a few examples. The size of the cloud-point to be processed generally proves a weakness, as dense or semi-dense clouds are generally impossible to be processed in real-time with graph-based algorithms.

Model based approaches are mostly based on the Random Sample Consensus (RANSAC), technique [14]. The procedure is based on fitting geometric primitive models, and grouping points according to their proximity to the models. The RANSAC approach itself has been widely studied [15,16] in fitting problems, and given an adequate model and initial seed produces accurate results robustly.

**Figure 2:**
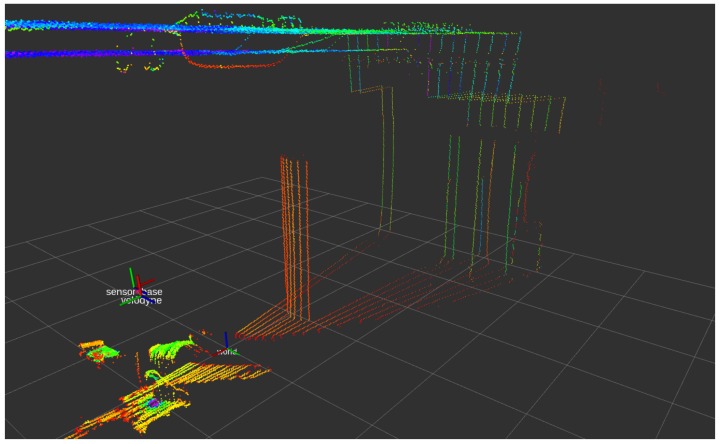
Characteristic sampling unevenness presented by most of the rotation-based LRF’s scanner, as the VLP-16 used in this work.

**Figure 3:**
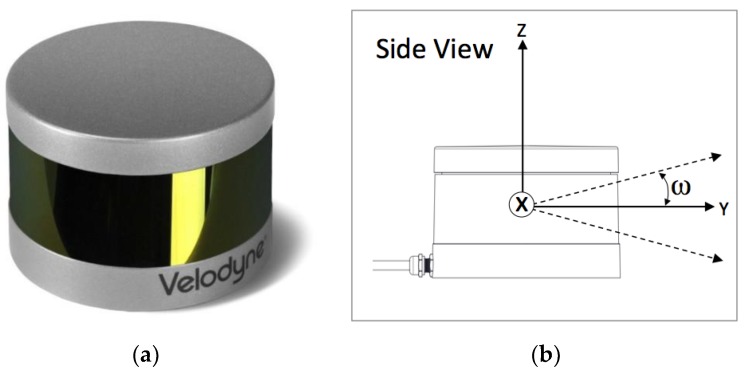
Sensor Velodyne^®^ VLP16: (**a**) appearance of the sensor; (**b**) sensor coordinate frame, with origin and scan width detail.

Note that most of the popular laser range finder (LRF) sensors present a characteristic unevenness in sampling density and distribution, as they generally work by performing single or multiple parallel scans by rotating the range-finder element. Because of this operation, the samples are quantized at some tens of coordinates along a limited subregion of the dimension/axis orthonormal to the scan plane, while the scan plane or half-planes are usually fully sampled, as seen in Figure 2. For example, the sensor used for this study, the VLP-16 (Velodyne^®^ is trademark of Velodyne LiDAR, Inc., San José, CA, USA) presents 16 scan lines distributed between +15° to −15° in azimuth, with 360° coverage each [17] (see Figure 3). This feature can affect the segmentation and positioning problem, especially in terms of accuracy depending on the relative orientation between the sensors and the objects, as it will be discussed.

For our problem, in order to detect a pipe generalized as a Straight Homogeneous Circular Cylinder (SHCC) robustly, in a real-time scenario with the limited computational power deployable in an UAV, to determine their pose, a RANSAC-based segmentation approach was chosen, using a state of the art implementation [9]. Thus, assuming that there is a cylindrical pipe, which can be described as a SHCC, *C*, and a coordinate frame centered in the sensor *L*, with TLδ denoting the homogenous transformation from a world origin *õ* to this LiDAR frame, the RANSAC process tries to fit a SHCC model into the point cloud. This point cloud is referenced with respect to *L*, with seven parameters of the model to be fit, namely: the coordinates of a support point for the axis of the SHCC in frame *L*, **p***_c_* = [*x_p_*, *y_p_*, *z_p_*], a vector denoting the direction of said axis **v***_c_* = [*x_v_*, *y_v_*, *z_v_*], and the estimated radius *r_c_*. A seed for the radius parameter can be provided, in the form of a range [*r_min_*, *r_max_*], with the RANSAC procedure trying to force that *r_c_* satisfies said range.

The RANSAC technique requires a procedure to build candidate models using a Minimum Sample Set (MSS). Although the minimal cardinality *k* of the MSS is *k* = 5 for the general case [18] of recovering a SHCC using samples from an Euclidean ℝ^3^ space, and *k* = 3 for the special case when all the sample lie in a same plane normal to **v***_c_* [19], the procedure implemented uses only *k* = 2 points, **p**_i_. Notice that this would generally be impossible, as the minimal general solution requires *k* = 5, per [18], but as our architecture treats the point cloud as a surface set to estimate the local curvature, additional data on the normal vector of the surface, **n***_i_*, is available for each **p***_i_*:

(1) $$p_{i}^{}=\left(x_{i},y_{i},z_{i}\right)$$

(2) $$n_{i}^{}=\left(n_{i}^{x},n_{i}^{y},n_{i}^{z}\right)$$

Thus, given two sample points **p***_i_* for *i* = [0,1], each one with its own normal vector **n***_i_* per Equations (1) and (2), and using geometrical properties of the dot and cross products, seen in Equations (3) through (6):

(3) $$w=n_{0}+p_{0}-p_{1}$$

(4) $$a=n_{1}\cdot n_{1}\;;\;b=n_{1}\cdot n_{2}\;;\;c=n_{2}\cdot n_{2}$$

(5) $$d=n_{1}\cdot w\;;\;e=n_{2}\cdot w$$

(6) g=a·c−b·b

it is possible to define the scale factors *sc* and *tc*:

(7) $$sc=\{\begin{array}{ccc} 0, & if & \left|g\right|<\varepsilon \\ \frac{\left(b\cdot e-c\cdot d\right)}{g}, & if & \left|g\right|\geq \varepsilon \end{array}$$

(8) $$tc=\{\begin{array}{ccc} d\cdot b, & if & \left(\left|g\right|<\varepsilon \right)\wedge \left(b>c\right)\\ e\cdot c, & if & \left(\left|g\right|<\varepsilon \right)\wedge \left(b\leq c\right)\\ \frac{\left(a\cdot e-b\cdot d\right)}{g}, & if & \left(\left|g\right|\geq \varepsilon \right) \end{array}$$

which are the solutions of the linear combinations to obtain the support point **p***_c_* and the director vector **v***_c_*, for the searched SHCC, per Equations (9) and (10). Notice that, for both *tc* and *sc*, when the value of *g* is below an arbitrarily small ε, it means that the respective normal vectors **n**_1_ and **n**_2_ are almost parallel, so an alternative way to obtain *tc* and *sg* is applied:

(9) $$p_{c}=p_{0}+n_{0}+sc\cdot n_{0}$$

(10) $$v_{c}=p_{1}+sc\cdot n_{0}-p_{c}$$

This method exploits the fact that the normal vectors generally contain enough information to describe the orientation of **v***_c_* by themselves, unless they are close to parallel. Once the full parametrization of the SHCC model is achieved, a validation test, using the radius priors, rejects incorrect and/or spurious candidate models, per Expression (11):

(11) $$r_{min}\leq r_{c}=\frac{\left\|v_{c}\times \left(p_{c}-p_{0}\right)\right\|}{\left\|v_{c}\right\|}\leq r_{max}$$

This formulation was used as part of the implemented RANSAC approach. While synthetic experimentation probed satisfactory, proof of concept tests showed that the application of this RANSAC procedure was vulnerable to the unequal distribution of samples along the different dimensions of the sensor frame *L*. This fact produces either false positives if the seed range for *r_c_* was set with wide margins; or failing to find a cylinder *C* with parameters fitting the SHCC model. In order to avoid this situation, an architecture to produce denser point clouds was developed, exploiting the assumption that odometry measurements of the movements or the multicopter would be available, so an approximation to TLδ is available.

**Figure 4:**
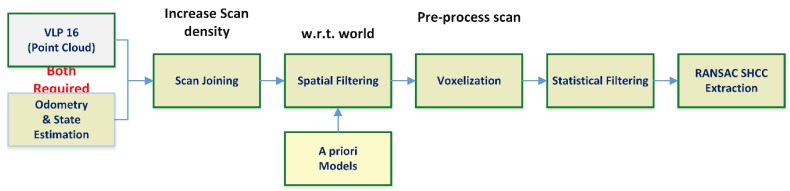
Initial architecture for the RANSAC-based detection and positioning of a SHCC in LiDAR point clouds data. Notice that due the unevenness of the LiDAR sampling, several point cloud were joined to produce denser scans. This requires very accurate odometry or large computational time, which was later proved a weakness to be dealt with.

The procedure (Figure 4) starts with a Scan Joining step, where two or more of the point clouds scans produced are combined to produce an assembled point cloud. This operation is performed exploiting the capabilities to store and operate several buffers of time-stamped transformations and frames provided by ROS [20,21]. 

Note that this procedure is entirely reliant in the accuracy of the transformation TLδ and the sensing capabilities of the multicopter to optimize its performance, as an ICP-like [22] procedure uses the transformation between the point clouds at different time instants as a seed. The main risk to this approach is correlated with the size of the assembled point cloud, as it grows linearly with the number of scans fused. If the assembled point cloud is larger than the size limit, which can be robustly solved in real-time, it again produces inaccurate model fittings or spurious detections. To avoid this, the point cloud is preprocessed to reduce the number of points considered in the RANSAC approach:

- In a first step, a geometrical based pass filter removes those points lying on regions, which can be predicted to appear in the cloud, but are known not to contain the target pipe. This includes shadows produced by the body of the UAV itself, and regions of the space determined as not relevant according to the pose or facing of the UAV, like the floor. This relies on previous knowledge and measurement from other sensors to determine the pose and facing of the UAV.
- In a second step, a voxelization filter is applied, reducing the size of the cloud. The voxel size can be adjusted considering that the nominal ranging accuracy of the LRF sensor used is known, and can be considered accurate, as seen in [17]. Note that if the voxelization greatly reduces the point cloud, it could mean that some strange body may be occluding the LiDAR by being too near to it.

The final step is a simple removal of statistical outliers based on the neighborhood of the data points. As the objective is detection and segmentation of the surfaces of an object, relevant points will be rarely pruned, as they are not isolated.Once the cloud has been filtered, the RANSAC procedure determines the model of a homogeneous circular cylinder described by an axis (a line with a support Euclidean point and direction vector) and a radius, by fitting the parametric model based on the neighbor surface normal of the data points.

This approach was tested in indoor environments, with a false positive detection rate below 0.7%, and a very accurate SHCC model parameter estimation. Anyway it presented two main weaknesses: firstly, the segmentation operation operated at an average rate of 0.73 Hz; and secondly, the indoor testbed used to simulate the odometry (estimated through motion capture within an *Optitrack*^®^ arena, [23]) produced an estimation with an accuracy beyond what it can be really expected during actual flight operations with on-board sensors. Introducing white noise into the odometry estimated with the motion capture system to simulate the actual accuracy that can be expected from real-time inertio-visual odometry approaches [24] produced a decrease in performance, with an average detection rate of 0.64 Hz. Still, the results obtained from testing this early architecture allowed to experimentally determine the processes and parameters needed to develop a faster approach.

**Figure 5:**
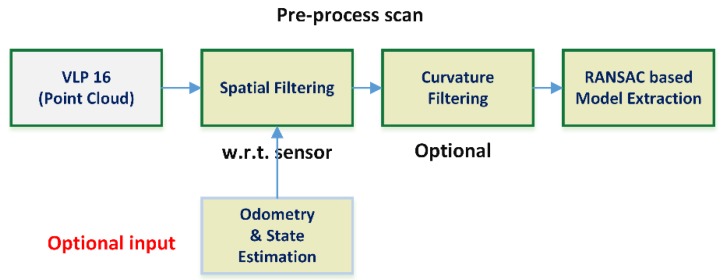
Optimized architecture for the RANSAC-based segmentation and positioning of SHCC in point clouds. All a priori knowledge is optional, although an initial radius estimation is strongly desirable as it is one of the main criteria available to reject spurious detections. The curvature filtering reduces the chances of fake positives when the initial radius is completely unknown, but requires heavy computational efforts.

This new lightweight architecture (see Figure 5) presents several differences over the initially tested: the cloud point joining process is removed, just like the statistical filter and the voxelization; and a new curvature-based filter is introduced. Most of the modifications were possible to introduce due to a better adjustment of the RANSAC parametric model and parameters, which was now able to detect the desired SHCC with single point clouds, avoiding the scan-joining step, as seen in Figure 4. This in turn removed the dependence on accurate odometry, with spatial filtering being generally done w.r.t. the sensor frame to remove the “shadow” of the UAV/rigid solid where sensor is attached. The statistical filter was removed as it was observed that it presented no relevant impact into the accuracy of the RANSAC procedure, neither to avoid fake positive nor improving accuracy. The voxelization process, though it had proved useful for dealing with dynamically sized cloud points, with the single point cloud approach it proved too expensive, as it is essentially a full resampling of the whole data.

## 4. Vision-Based Detection and Pose Recovery of a Cylindrical Pipe

**Figure 6:**
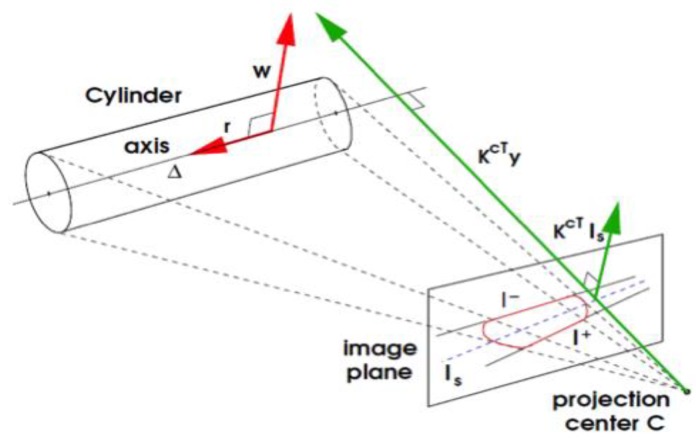
Projection of the apparent contours of a SHCC modelling a pipe in the image plane, with the camera projection center and the pose coordinates (courtesy of Doignon et al. [29]).

One of the main physical characteristics of pipes and tubes, in terms of vision-based perception and image, is the apparent contour, i.e., the edges presented: even when they present similar hue and texture as the background, the geometry of a pipe, as a SHCC, is noticeable (see Figure 6). Another important characteristic that can be usually detected and tracked is the material texture. Nevertheless, this saliency in terms of texture with respect to the rest of the environment may prove unreliable, as its detection can be largely affected by shadows, dynamic lighting, and other visual artifacts. These issues can be dealt with through computer vision techniques, but generally imply computationally expensive procedures, unsuitable for UAV deployment.

### 4.1. Pose Recovery

Several vision-based approaches have tried to solve the pose estimation problem for cylinders from monocular images. In [25], several methods to estimate linear and quadratic primitives through analytic procedures are presented, focusing in the perspective inversion approach. In [26], a multistep process localizes each of the cylinder axis using a priori knowledge about the cross-sections projection, as described in [27], and use them to localize the cylindrical surface in the camera coordinate frame. More recently, in [28], the metric reconstruction of surfaces of revolution (SOR) was addressed combining the apparent contour and captures of cross-sections. Some of the geometrical properties and formulations described in [28] were also used in work by Doignon [29]. Later works [30] have proposed solutions based in non-linear Levenberg-Marquardt optimization, though they tend to rely in multiple views and iterative solutions.

In [29], Doignon et al. present a pose recovery method for SHCC from the apparent contour in a single image. A closed-form solution to determine the pose between the axis of the SHCC and the camera scaled by the radius in Plücker coordinates [31] is given. This is achieved by formulating a matrix representing the degenerate quadratic defining the cylinder, which can be annotated as Plückerian coordinates of the symmetry axis (see Figure 6). This formulation can be used in a conic-based pose fitting method, which can determine the pose exploiting the relations between the perspective projection and the pose parameters.

Thus, in order to determine the Plückerian coordinates (**r***_e_*, **w***_e_*) of the axis, this method assumes that the apparent contour contains at least 2 segments, **a** and **b**, with known extrema annotated in homogeneous coordinates, namely (**p***_a_*_0_, **p***_a_*_1_) and (**p***_b_*_0_, **p***_b_*_1_) respectively. This parametrization is derived from the pixel coordinates of a point **p** = [*u v*]^T^ in an image, per Equation (12):

(12) $$\begin{array}{l} p_{a0}=\left[\begin{array}{ccc} u_{a0} & v_{a0} & 1 \end{array}\right]\;;\;p_{a1}=\left[\begin{array}{ccc} u_{a1} & v_{a1} & 1 \end{array}\right];\\ p_{b0}=\left[\begin{array}{ccc} u_{b0} & v_{b0} & 1 \end{array}\right]\;;\;p_{b1}=\left[\begin{array}{ccc} u_{b1} & v_{b1} & 1 \end{array}\right]. \end{array}$$

These segments, **a** and **b,** are contained in lines **l***_a_* and **l***_b_*, whose homogeneous coordinates are obtained through cross product of the homogeneous coordinates of the segment extrema:

(13) $$l_{a}=p_{a0}\times p_{a1}\;;\;l_{b}=p_{b0}\times p_{b1}$$

In order to compute the degenerate conic to solve, the apparent contour lines **l***_a_* and **l***_b_* must be normalized into **l***_an_* and **l***_bn_*:

(14) $$l_{an}=l_{a}\frac{1}{\left\|l_{a}\right\|}\;;\;l_{bn}=l_{b}\frac{1}{\left\|l_{b}\right\|}$$

The camera intrinsic parameters are used to compose the intrinsic calibration matrix *K*. This matrix contains the principal point coordinates in mm, (*x*_0_, *y*_0_); the focal length of the optics in terms of pixel size, for the horizontal and vertical axes, α*_x_* and α*_y_*; and the skew *s*

(15) $$K=\left[\begin{array}{ccc} \alpha_{x} & s & x_{0}\\ & \alpha_{y} & y_{0}\\ & & 1 \end{array}\right]$$

With this data, a degenerate conic equation relating the parametrization of the apparent contour of the cylinder with the calibration matrix for the given image can be obtained through Equation (16):

(16) $$C=K^{T}\times \left(l_{an}\times l_{bn}{}^{T}+l_{bn}\times l_{an}{}^{T}\right)\times K$$

The conic can be solved through singular value decomposition [32] of matrix *C*, in the form

(17) (U,D,V)=svd(C),

which produces a direct estimation of the director vector of the axis of the SHCC, **r***_e_*, as the last column of the left singular vectors matrix *U*

(18) $$r_{e}={\left[\begin{array}{ccc} U_{2,0} & U_{2,1} & U_{2,2} \end{array}\right]}^{T}$$

In order to estimate **w***_e_*, a vector describing the direction between the camera optical center and the nearest point of the axis, **y***_e_*, using the second column of the singular vector matrix, scaled using the major singular values to compute *σ*, (Equation (19))

(19) $$\sigma =\sqrt{1-\frac{D\left(1,1\right)}{D\left(0,0\right)}}$$

(20) $$y_{e}=\sigma \times {\left[\begin{array}{ccc} U_{1,0} & U_{1,1} & U_{1,2} \end{array}\right]}^{T}$$

Once **y***_e_* is found, a scaling term *n_w_* is found, computed according to the radius of the SHCC, *r_c_*, according to Equation (21). Solution for the second vector of the Plückerian coordinates, **w***_e_* is then achieved per Equation (22). Notice that as **w***_e_* is orthonormal to both the director vector of the axis of the SHCC, **r***_e_*, and the and director vector from *C* to the axis through the shortest path, **y***_e_*, it is obtained through a normalized cross product scaled with *n_w_* to force the scale of *r_c_*

(21) $$n_{w}=r_{c}\cdot \sqrt{1+\frac{1}{\left(1-y_{e}{}^{T}\cdot y_{e}\right)}}$$

(22) $$w_{e}=n_{w}\times \frac{y_{e}\times r_{e}}{\left\|y_{e}\times r_{e}\right\|}$$

This solution was implemented into a ROS node to visually determine the pose of the pipe, as the closed-form solution described meant that the procedure could achieve real-time performance, because only a singular value decomposition operation was required to solve the optimization part of the method. Tests with synthetic datasets for apparent contours showed results consistent with those described in the original work. Indoor experiments were also successful, producing average relative error below 3.5% for depth estimation. Still, when the camera optical axis and the pipe axis become close to parallel, which constitutes a degenerate configuration, the method becomes inconsistent.

### 4.2. Apparent Contour Extraction

Several approaches were developed in order to extract the apparent contour of a pipe. A simplistic solution based in the Hough transform [33] was initially developed, where all the straight lines in a region of interest are detected and studied. During the initial indoor testing the probabilistic Hough transform based on Canny edge detector [34] with Otsu’s threshold [35] proved enough to achieve consistent binarization and edge detection (note that Canny is still widely known as an *optimal detector* [36]). A set of possible candidates to apparent contour was chosen, finding pairings of lines. In order to find initially the apparent contour candidates, a five-step procedure was followed:

- Filter segment lines shorter than *l_min_*.
- Group segments by general orientation, i.e., most likely to be horizontal or vertical.
- Pair segments by angle and length similarity.
- Select candidates from those pairs where no other edge is found between them.
- Candidate validation step using priors.

A priori knowledge was used during the final validation step to choose the apparent contour candidate to use in the method described earlier to recover the pose. This knowledge was introduced as geometric/model restrictions (i.e., approximately known orientation or position of the pipe), or through a human machine interface (HMI). Notice that using HMI knowledge to obtain priors required using accurate odometry to transform the prior knowledge to the relevant coordinate frame of the camera. To add consistency to the method, once an apparent contour has been found and validated, a visual servoing tracking method [37] searches for it in successive frames, and only when there are inconsistencies the full detection is performed.

**Figure 7:**
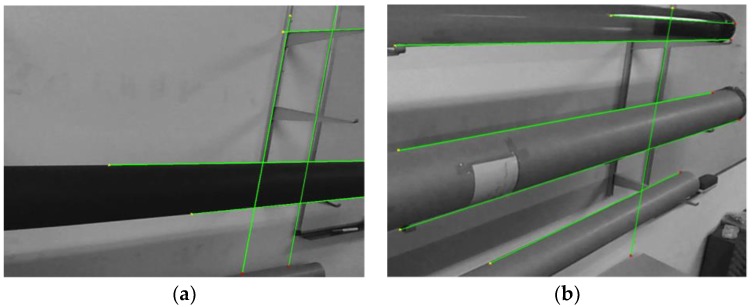
Two samples of Hough transform pipe detection. Random elements can easily present straight edges in structured environments, so the procedure needs to solve ambiguities exploiting prior data or image processing: (**a**) two pair of lines detected which can produced two equally strong apparent contour candidates, discrimination can be only made through a priori knowledge about the model or time consuming computer vision techniques to enable scene interpretation; (**b**) the reflection in the top pipe may produce a spurious apparent contour, disrupt pose recovery, while shadows in the bottom pipe avoid determining a correct apparent contour.

This implementation, including pose recovery, produced robust results in indoor environments in terms of detection, but presented poor performance around 8.64 Hz, while still being affected by multiple challenging issues in terms of computer vision (see Figure 7). 

A small battery of outdoor tests further revealed some critical weaknesses. Firstly, the global binarization process was not able to properly detect edges under natural uncontrolled lighting, especially when multiple/ambient light produce diffused shadows; the implicit assumption of presenting features similar to a bimodal image taken in the indoor case to use Otsu’s thresholding was not useful in an uncontrolled environment. Additionally, the indoor structured environments presented more easily identified contours, usually presenting stronger edges with approximately known size and structure; thus able to be detected and identified with our assumed model. Finally, in the outdoor operation, the frame to frame contour tracking was unable to track the contour consistently, requiring to reintroduce prior knowledge in the case of the HMI.

**Figure 8:**
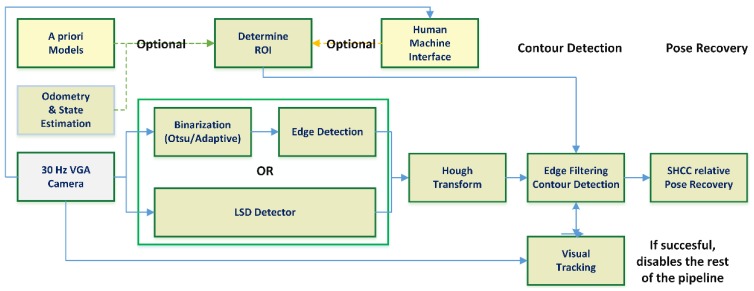
Resulting vision-based architecture for detection and pose recovery of pipes. A priori data obtained through a HMI (where the human chooses the edges of the apparent contour) or known priors about the pipe and the odometry estimation is required to add robustness to the contour determination process. The visual tracking, frame to frame, of the edges composing the apparent contour can speed up the process notably, disabling most of the visual pipeline.

A modified approach substituted the global binarization with two different local adaptive binarization approaches [37], but the performance achieved was too low to be useful, with 2.34 Hz on average at 640 × 480 pixels. In the end, the full binarization with Canny edge detection was removed in favor of introducing a line segment detector (LSD [38]). This final architecture, seen in Figure 8, improved the performance of the approach, working at an average 21.4 Hz, but still presented an unreliable contour detection step, as it is discussed further in the results section.

## 5. Integrated LiDAR Segmentation and Vision-Based Pose Recovery

Earlier sections have discussed work developed with each of the available sensors in order to solve the problem of detection and pose recovery of a pipe with known radius. Of the studied approaches, using LiDAR and vision respectively, each one presented its own weaknesses and strengths. Our study showed that each of the approaches was stronger at one of the steps and noticeable weaker at the other task: the LiDAR registration procedure achieved great robustness at the detection and segmentation task, while the vision based pose recovery presented great accuracy at higher rate, but with very weak detection results. These results led to the development of a combined approach to exploit the best features provided by each sensing technology.

The integrated method solves the problem in two different steps, working at different speeds with different sensors. Firstly, a RANSAC-based segmentation step, as described earlier, uses the point cloud data provided by the VLP 16 LiDAR to fit the SHCC model into the environment surrounding the UAV. This process works at an average 4.3 Hz, with an accuracy presenting dependencies w.r.t. the material and texture of the pipe to be detected, and especially to the relative position between the pipe axis and the sensors, as will be discussed in Section 6. Once an estimation of the pipe axis pose is available, this line is projected into the camera plane using the projection matrix of the calibrated camera sensor [39]. This allows determining a well bounded ROI to search for line segments in the image, reducing the computational load without compromising the robustness of the approach, and use a robust seed to discriminate the apparent contour from the set of lines produced by the Hough transform.

**Figure 9:**
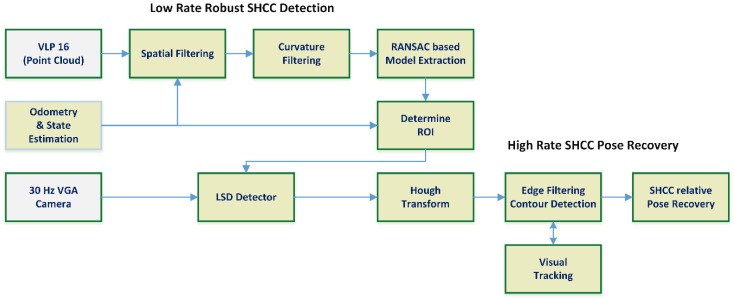
Integrated architecture combining the developed LiDAR and vision based pipelines. The initial detection of the pipe is provided by the LiDAR RANSAC segmentation, providing very robust detection with average accuracy positioning. Using data from the FMU odometry or any other state estimation process available (e.g., any SLAM approach, visual odometry, etc.) the estimated pose of the pipe is used as a prior in the visual pipeline, simplifying the visual detection problem. Thus, the greater accuracy of the visual pose recovery can be exploited.

Figure 9 shows the architecture diagram for the combined approach. The first row shows the LiDAR-based segmentation pipeline, starting with the point cloud data obtained from the VLP 16 sensor, and following the process shown in Figure 5, which provides robust detection of the pipe and an initial pose estimation. In the second row, the step to convert the initial pose estimation produced by the LiDAR into a prior for the visual pose recovery is shown. Note that in order to be able to use pose estimated by the RANSAC-based cylinder segmentation, an estimation of the state and odometry of the UAV/sensors rigid body is required, as the LiDAR segmentation and visual positioning pipelines work at different rates. Under these conditions, authors cannot assume that the global position of the UAV/sensors rigid body will not vary and use the relative pose between the LiDAR detected cylinder and the UAV directly (as the frequency achieved is around 4.5 Hz the delay is around ~0.23 s). We can assume that the odometry estimation provided by the FMU (as described in Section 2, see Figure 1) will be locally accurate to transform the estimated line parameters into current camera coordinates. This data is then used in the *third row* of the architecture diagram, which details the visual pipe segmentation and pose recovery. Notice that although some measure of scene registration is still performed, the visual pipeline has been modified to use the data from the LiDAR detected pipe as a prior, so the processes and architecture described in Section 4.2 are simplified and the apparent contour detection rate is greatly improved. These modifications remove the need for human feedback or accurate pipe priors; the only required that is the cylinder radius, with the pose recovery process remaining largely the same once the apparent contour is determined.

## 6. Experimental Validation

The proposed approach has been validated with experimental data. Each of the different techniques and architectures was tested using the relevant sensors and ground truths. 

**Figure 10:**
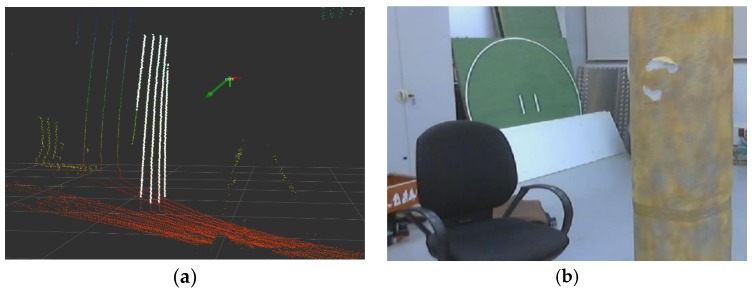
Samples of one of the indoor experimental sequences captured: (**a**) visualization of the VLP16 point cloud, with and RGB axis frame denoting the rigid sensor frame pose, and those point pertaining to the detected SHCC plotted in white; (**b**) view from the camera rigidly attached to the sensor frame, the elements seen (pipe, chair) can be also observed in figure **a**.

The experiments were performed over sequences captured (see Figure 10) through software provided by the ROS middleware. The software developed was integrated into the ROS framework, and tested in a i7 laptop, at 2.5 GHz, running ROS Indigo over Ubuntu Trusty Tahr.

### 6.1. Experimental Hardware Setup

**Figure 11:**
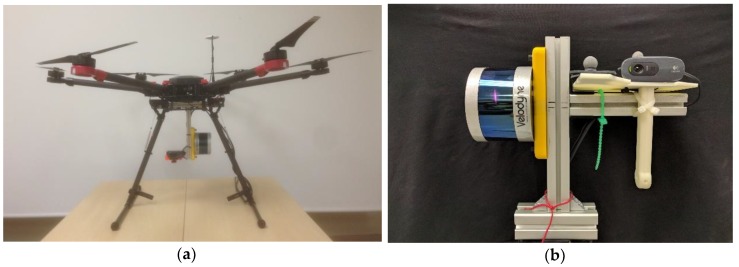
Hardware Prototypes: (**a**) early prototype UAV hexacopter deploying the sensor setup considered in this work; (**b**) hand-held sensor rigid body for experimentation in flight-denied areas (indoor laboratories, etc…).

Two different hardware setups have been used to capture sequences tested with the developed techniques. Firstly, a multicopter drone platform, used as concept test, to check viability of flight with the increased weight and impact of vibrations and other disturbances introduced. An early image of the prototype target platform to deploy the developed software can be seen in Figure 11a. The robotic UAV platform is largely based on a commercial hexacopter UAV (DJI^®^ is a trademark of DJI Corp., Shenzhen, China). A second hardware setup was developed in order to test and validate the different techniques developed without having to perform real flights, a standalone rigid frame was built to deploy the sensors, and operate them manually in indoor environments (see Figure 11b). Working with the hand held sensor frame allowed us to easily study singular configurations and other cases of interest, and also permitted testing the approaches with data obtained inside and indoor motion capture system, providing accurate ground truth.

In both setups, the UAV and the handheld frame, the Y axis of the VLP 16 was aligned parallel to the visual axis of the camera (commonly Z in camera frame according to literature). This meant that although there is no actual difference between X and Y axes in terms of LiDAR sensing capability, as during the capture the camera was pointed towards the pipe, the Y axis of the LiDAR became the depth from the sensor to the pipe, while the X axis mapped the pan or side-scrolling movements. Thus, during the results discussion, those discussions referred to the Y axis of the LiDAR are actually related to the depth between pipe and sensor.

### 6.2. LiDAR Detection and Positioning Results

In order to evaluate the LiDAR segmentation robustness and accuracy several indoor tests were performed locating a vertical 0.5 m diameter pipe; using an accurate ground truth produced with an Optitrack® system (Natural Point Inc., Corvallis, OR, USA). Several set of sequences, performing multiples passes of similar trajectories were captured and used to test the algorithms. The first validation step was finding if the lightweight architecture without scan joining could achieve the same robustness, and how much better performance could be achieved. It was determined that the false positive rate was almost negligible for both (see Table 1), but at the same time, avoiding the scan joining step reduced greatly the computational effort. This is noticeable not only in the joining and pre-processing phases, but also in the RANSAC step, as the number of average points introduced into the RANSAC method went down from an average of 19k to 8.5k, thus greatly alleviating the computational costs. The impact is evident in the average frame rates achieved by each method.

**Figure 12:**
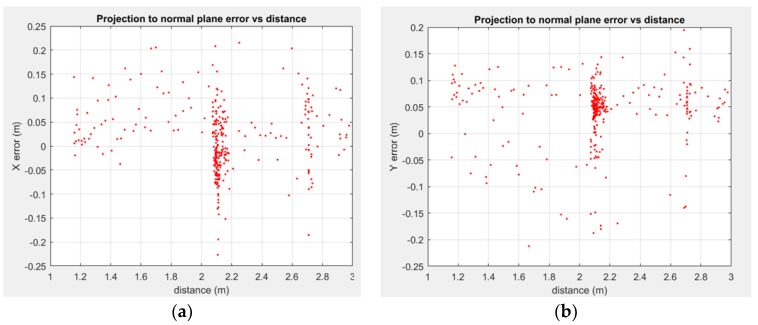
Distance between LiDAR and SHCC vs. position error in the plane XY of the LiDAR: (**a**) X position error in the XY plane of the LiDAR at the pipe axis intersection; (**b**) Y position error in the XY plane of the LiDAR at the pipe axis intersection.

**Figure 13:**
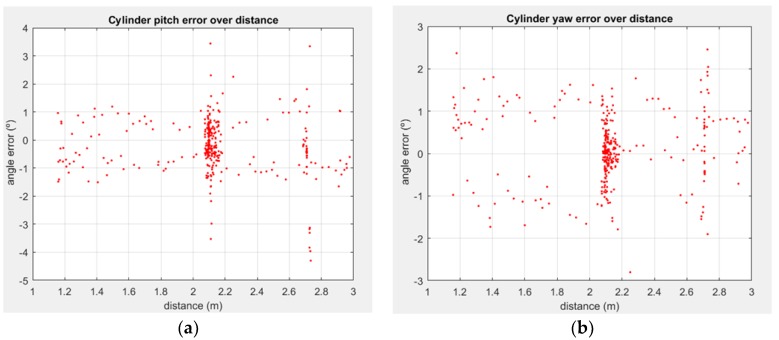
2 D.o.F. orientation between estimated SHCC axis and actual vertical pipe, assuming as roll rotation around the pipe axis which extents along the Z axis of a coordinate frame: (**a**) pitch error, observed as the projected angle in a XZ plane containing the pipe axis; (**b**) yaw error, as a projected angle in a YZ plane containing the pipe axis.

The impact of the distance and orientation between the pipe and the sensor was studied using the ground truth from the motion capture system. Figure 12 and Figure 13 show the impact of distance in position and orientation estimation, respectively, for one of the experimental sequences. In said experiment the rigid sensor frame was set a 3.5 m distance from the pipe, then the distance was closed until ~1 m, and then moved away from it again. At around 2.10 m, the sensor frame was rotated in several axes, with multiple roll rotation around the line joining the LiDAR and the pipe axis. 

It is noticeable how in all the degrees of freedom the error is well bounded, and when studying the 2.10 m point, as the most sampled distance, the error tends to follow a normal-like distribution (with a slight bias in the depth estimation, noted as Y axis with respect to plane XY plane of the LiDAR, per Figure 3b). The study of the orientation error with respect to the distance shows (Figure 13) that it is well bounded around 1° for one axis, at Figure 13a, with slightly more disperse results for the angle in the YZ plane (Figure 12b). Notice that this angle is correlated with depth perception, and as such, it presents a slightly greater error, as it is noticeable in Figure 13b.

**Figure 14:**
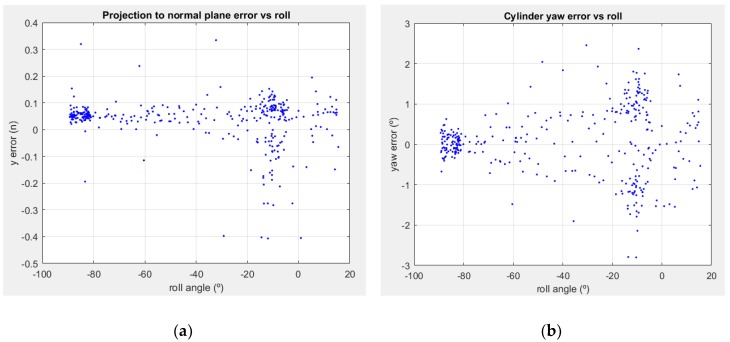
Depth positioning and orientation error vs. sensor roll around LiDAR Y axis: (**a**) position error in pipe axis interception against the XY plane of the LiDAR, in the same XY plane; (**b**) yaw error, as a projected angle in a YZ plane containing the pipe axis.

The study of the sensibility of the SHCC estimation with respect to the orientation of the sensor showed a strong correlation between the roll along the Y axis of the sensor itself, and the *depth* related position and orientation components. The relevant results are shown in Figure 14a,b, respectively. The low dispersion cluster with very low errors around 90° were produced, both for position and orientation in short distances, below the 2 m marks, with the scan planes orthonormal to the floor and aligned to the pipe axis. The other big clusters are near horizontal orientations of the sensors, and present a much wider dispersion. This phenomenon was produced by the different detection rates, affected both by distance and orientation. As such, the approximately vertical orientation of the sensor, with scan lines almost parallel to the pipe, produces much more accurate results if the distance is close enough so that enough scan lines will hit the pipe, enabling detection of the SHCC through RANSAC. If the distance crosses the 2 m mark, the accuracy drops slightly, but it is also prone to fail to find the SHCC in the point cloud.

On the other hand, when the sensor was horizontal, presenting scan lines orthonormal to the pipe, the detection range was much wider. This led to a greater dispersion of the error measurements, as these measurements were produced in the whole range between 1 m and 3.5 m.

### 6.3. Vision Based Countour Detection and Pose Recovery

**Figure 15:**
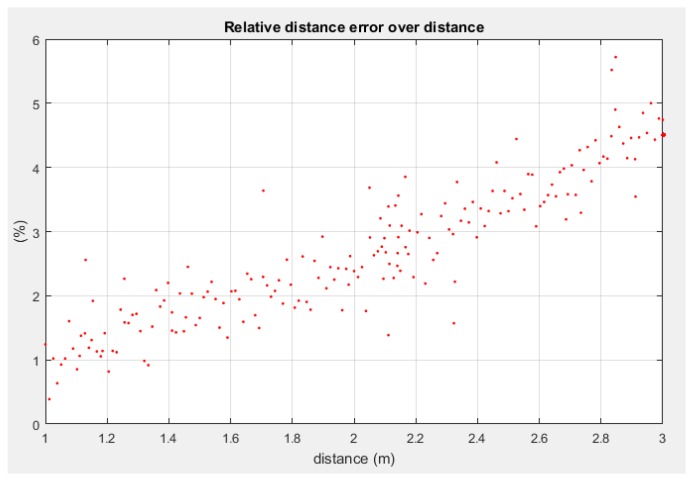
Pose recovery accuracy against distance, measured as the relative error between the Euclidean norm of the shortest distance from the camera centre to the estimated axis and the real axis.

The vision based approach was tested with the same indoor sequences, and some other outdoor sequences, which lacked ground truth or LiDAR information. The accuracy of the results obtained, in terms of pose recovery, was worse than those reported by [29], with an average 4.2% relative error in depth to the axis estimation. The relative error ε*_r_* for each view was measured as the ratio between the absolute difference between the Euclidean norms of **q***_e_* and **q***_m_* and the actual norm of the distance:

(23) $$\varepsilon_{r}=\frac{\left|\left\|q_{e}\right\|-\left\|q_{m}\right\|\right|}{\left\|q_{m}\right\|}$$

where **q***_e_* is the shortest segment between C and the estimated axis line (**r***_e_*, **w***_e_*), and **q***_m_* is the same segment parametrized through values experimentally measured with the motion capture system. Figure 15 shows how the pose recovery relative error, in terms of norm of the recovered pose, grows with distance. This can be expected from this kind of approaches, as they are affected by Abbe’s error and the loss of perceived relative accuracy as the pixel/distance ratio decreases.

In Table 2 statistics on the different approaches studied are displayed, showing the accuracy of the contour detection step, in terms of false positive rate (i.e., instances where no contour is present, or an incorrect contour is detected), and a general estimation of the performance of each method as the average rate achieved. These tests exclude deliberately using knowledge introduced through a HMI, as that method just delegates the apparent contour detection to the human component, and only uses approximate a priori knowledge about the pipe (i.e., the general orientation and initial distance), and an odometry estimation.

The purely edge attribute-based methods (**a**, **b** and **c** in Table 2) tested have been found unable to solve the general pipe contour detection problem in a fully satisfactory way, as seen in the high spurious detection rates. Several vision-based approaches could be used to improve the results, ranging from segmentation technique, probably including texture analysis to improve detection of common pipe materials, to state of the art deep learning/convolutional neural networks, which combine probabilistic detection and segmentation. However, all these approaches, and most of those that could have a noticeable impact in the detection rates tend to be computationally heavy; and at the same time, the performance achieved in a commercial laptop i7 processor is already below the desirable threshold for most of the approaches, while the computational power deployable in a multicopter UAV is roughly equivalent. These facts remove the pure vision-based approach to pipe contour detection onboard an UAV as an option, leading to the integrated LiDAR and vision method.

The method proposed integrating both LiDAR and vision (entry **d** in Table 2) presents the best detection rate, as the apparent contour is detected using as support the actual estimation of the pipe according to the LiDAR-based segmentation (which presented spurious detection rates below 1%). It is interesting how the performance of the vision based pipeline of the integrated method is slightly lower than that of the equivalent technique (entry **c** in Table 2) without LiDAR, though the most probably cause is the need added layer introduced by the data sharing and conversion between frames.

## 7. Conclusions

A methodology to accurately detect and recover the pose of a pipe (or any other cylindrical structural element) with respect to a robotic multicopter UAV has been developed. Initial studies tried to determine which of the available sensor devices, namely, monocular vision cameras or LiDAR, could provide a better solution to the detection and positioning challenges. These tests showed that none of the single-sensor solutions developed could provide an all-encompassing satisfactory solution. The LiDAR detection and positioning solutions were implemented based in RANSAC approaches, with two different developed architectures: one based in single LiDAR scan processing, and another one base on joining multiple LiDAR scans. The single scan architecture proved to be functionally as accurate as the approach with multiple scan joining, but presented a fivefold increase in performance measured as rate. This approach achieved very robust detection, with negligible false positives, but at a slow rate with average accuracy.

The visual pipelines developed were based in the pose recovery described in [29]. This required the detection of the apparent contour of the pipe, which proved to be a hard to solve challenge. As the more powerful and complex computer vision approaches are not deployable into UAV due computational budget constraints, several edge-based methods were proposed and studied, with different degrees of success. The most successful unsupervised approach offered better results than the LiDAR approach in terms of pose recovery accuracy and speed, but with poorer detection rates.

Thus, the integrated solution proposed uses the LiDAR to detect robustly the presence of the pipe and to produce an approximate estimation of its position, which in turn is projected into the image to use it as a seed to improve visual detection of the pipe. Once the pipe has been detected in the image, the apparent contour is extracted and used to recover the pose of an SHCC, considered the geometrical model of the pipe.

All the proposed methods have been tested with experimental data acquired in a motion capture testbed, which provided the ground truth for a rigid frame deploying the sensors used, in a configuration analogous to the one that could be found in and UAV. Additional vision−only sequences, captured with an actual multicopter, were used to test the vision−based approaches as the differences between indoor and outdoor environments greatly influence their performance.

## Figures and Tables

**Table 1:** RANSAC-based segmentation of a SHCC in point clouds, “joining scans vs. single scan”.

Method	Initial Size	RANSAC Size	Avg. Rate	False Positives
Figure 3 Architecture	~60k points	19k avg. points	0.73 Hz	0.71%
Figure 4 Architecture	~30k points	8.5k avg. points	3.94 Hz	0.73%

**Table 2:** Detection accuracy and performance of the visual detection of pipe apparent contours.

Contour Detection Method	Indoor Error Rate ^1^	Outdoor Error Rate ^2^	Avg. Rate
Canny + Hough Transform (**a**)	23.45%	48.71%	8.64 Hz
**a** + Adaptive Binarization (**b**)	17.29%	33.56%	2.34 Hz
LSD + Hough Transform (**c**)	19.42%	29.38%	21.4 Hz
**c** + LiDAR based priors (**d**)	1.32%	- ^3^	19.78 Hz

^1,2^ False positive rate (i.e., a contour not pertaining to the pipe or presenting wrong fitting is found). ^3^ No outdoor data with LiDAR and image is available.
